# *PRPF3*-Associated Autosomal Dominant Retinitis Pigmentosa and *CYP4V2*-Associated Bietti's Crystalline Corneoretinal Dystrophy Coexist in a Multigenerational Chinese Family

**DOI:** 10.1155/2017/4156386

**Published:** 2017-08-07

**Authors:** Xiaohong Meng, Qiyou Li, Hong Guo, Haiwei Xu, Shiying Li, Zhengqin Yin

**Affiliations:** ^1^Southwest Hospital and Southwest Eye Hospital, Third Military Medical University, Chongqing 400038, China; ^2^Key Lab of Visual Damage and Regeneration & Restoration of Chongqing, Chongqing 400038, China; ^3^Department of Medical Genetics, Third Military Medical University, Chongqing 400038, China

## Abstract

**Purpose:**

To characterize the clinical and molecular genetic characteristics of a large, multigenerational Chinese family showing different phenotypes.

**Methods:**

A pedigree consisted of 56 individuals in 5 generations was recruited. Comprehensive ophthalmic examinations were performed in 16 family members affected. Mutation screening of *CYP4V2* was performed by Sanger sequencing. Next-generation sequencing (NGS) was performed to capture and sequence all exons of 47 known retinal dystrophy-associated genes in two affected family members who had no mutations in *CYP4V2*. The detected variants in NGS were validated by Sanger sequencing in the family members.

**Results:**

Two compound heterozygous CYP4V2 mutations (c.802-8_810del17insGC and c.992A>C) were detected in the proband who presented typical clinical features of BCD. One missense mutation (c.1482C>T, p.T494M) in the PRPF3 gene was detected in 9 out of 22 affected family members who manifested classical clinical features of RP.

**Conclusions:**

Our results showed that two compound heterozygous CYP4V2 mutations caused BCD, and one missense mutation in PRPF3 was responsible for adRP in this large family. This study suggests that accurate phenotypic diagnosis, molecular diagnosis, and genetic counseling are necessary for patients with hereditary retinal degeneration in some large mutigenerational family.

## 1. Introduction

Retinitis pigmentosa (RP) (MIM 268000) is the most common form of hereditary retinal degeneration (HRD), with a worldwide prevalence of 1 in 4000 [1]. The disease can be inherited in an autosomal recessive (AR), autosomal dominant (AD), or X-linked manner [2]. Autosomal dominant RP (adRP) is the most common form of RP and typically begins with night blindness in the early teens, followed by progressive loss in the peripheral visual field, subsequent loss of vision, and eventually legal blindness. To date, mutations in 22 genes have been associated with adRP (RetNet: http://www.sph.uth.tmc.edu/retnet/sum-dis.htm, last updated November 16, 2016), of which five genes have been reported in Chinese adRP patients [3–7].

Bietti's crystalline corneoretinal dystrophy (BCD) (MIM 210370) is an autosomal recessive retinal dystrophy that is characterized by numerous tiny glistening yellow-white crystals that are scattered at the posterior pole of the retina, progressive atrophy of the retinal pigment epithelium (RPE), and choroidal sclerosis. Patients with BCD are usually present in the 2nd or 3rd decade of life and progress to legal blindness by the 5th or 6th decade [8]. Mutations in the *CYP4V2* gene (MIM 608614) are associated with BCD [9]. BCD is relatively common in the East Asian populations, especially in Chinese and Japanese populations [9–18].

BCD and RP are considered as two different types of retinal dystrophies with distinct clinical courses and features during its early stage. However, the fundus features at the later stage of BCD are occasionally similar to a severe form of RP. The interaction or coexistence of the two clinical phenotypes thus requires further elucidation. In the present study, we distinguish the inheritance patterns, clinical phenotype, and molecular genetic characteristics of the patients in a large, multigeneration Chinese family with RP and BCD.

## 2. Methods

### 2.1. Pedigree

A pedigree consisted of 56 individuals in 5 generations was recruited. The Ethics Review Board of the Southwest Hospital (Chongqing, China) approved the research protocol (number 2012-11), which adhered to the tenets of the Declaration of Helsinki, and informed consent was obtained from all participants.

**Figure 1:**
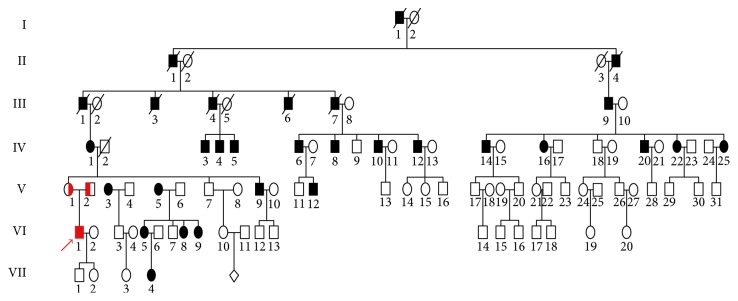
Pedigree plot. The proband is indicated by an arrow. One affected patient with red solid box showed clinical findings compatible to the diagnosis of Bietti's crystalline corneoretinal dystrophy (VI:1), and the other affected members with black solid box presented clinical features of retinitis pigmentosa. Males and females are represented by squares and circles, respectively. Filled symbols: affected members; open symbols: unaffected members.

The proband (Figure 1, VI:1) was initially presented to our medical institution for genetic counseling based on the observation that most of the family members developed night blindness and visual loss and even complete blindness, resulting in an inability to work. This five-generation family from Southwest of China was assessed in terms of RP and BCD. Thirty-nine participants were ascertained at the Southwest Eye Hospital, Southwest Hospital, Chongqing, China (Figure 1). No consanguineous marriage in the family was declared.

The proband presented clinical features that were compatible with a diagnosis of BCD (VI:1), and the family members were subsequently evaluated. Twenty-two living individuals in the family had the clinical features of RP and presented similar symptoms of night blindness and progressive reduction in their field of vision. The RP phenotype followed an autosomal dominant pattern of inheritance in this pedigree (Figure 1).

Thirteen affected individuals (III:9, IV:1, IV:3, IV:5, IV:6, IV:10, IV:12, IV:14, IV:20, V:3, V:9, V:12, and VI:1) and twenty-six unaffected family members (Table 1) underwent examination, including best-corrected visual acuity testing with the Snellen vision chart, fundoscopy, slit-lamp biomicroscopy, spectral domain optical coherence tomography (SD-OCT, Spectralis OCT, Version 6.0; Heidelberg Engineering, Germany), full-field electroretinogram (FERG), and multifocal electroretinogram (mfERG). For the ages 6 months, 1, 2, 3, and 4 years old, the visual acuity was assessed using Teller acuity cards and then converted into Snellen vision chart.

### 2.2. Mutation Screening

Genomic DNA was extracted from peripheral blood samples of 39 family members (Table 1) using a QIAamp DNA Blood Midi Kit (Qiagen, Hilden, Germany) following the manufacturer's standard procedure. All coding exons and intron-exon boundaries of the *CYP4V2* gene were amplified by polymerase chain reaction (PCR) using primers described by Li et al. [9]. The PCR products were subsequently purified with a TIANgen Mini Purification Kit (Tiangen Biotech Co. Ltd., Shanghai, China) and sequenced by Sanger sequencing with an ABI BigDye Terminator Cycle Sequencing Kit v3.1 (Applied Biosystems (ABI), Foster City, CA). *CYP4V2* sequencing was performed in eight patients (III:9, IV:1, IV:6, IV:12, V:1, V:12, VI:1, and VI:2), and the detected mutation was further screened in 12 affected family members and 11 unaffected members.

Next-generation sequencing (NGS) was then applied to two affected family members with RP (III:9 and IV:1), who did not have *CYP4V2* mutations, then to identify disease-causing variants in 47 RP-related genes including the *PRPF31*, *CRB1*, *PRPF8*, *CA4*, *TULP1*, *PRPF3*, *ABCA4*, *RPE65*, *EYS*, *CERKL*, *NRL*, *FAM161A*, *FSCN2*, *TOPORS*, *SNRNP200*, *SEMA4A*, *PRCD*, *NR2E3*, *MERTK*, *USH2A*, *PDE6B*, *PROM1*, *KLHL7*, *PDE6A*, *RGR*, *CNGB1*, *IDH3B*, *SAG*, *GUCA1B*, *CNGA1*, *BEST1*, *TTC8*, *C2orf71*, *ARL6*, *IMPG2*, *PDE6G*, *ZNF513*, *DHDDS*, *PRPF6*, *CLRN1*, *MAK*, *CDHR1*, *FLVCR1*, *RLBP1*, *SPATA7*, *AIPL1*, and *LRAT* genes. The detected variants in NGS were validated by Sanger sequencing and screened in other 6 affected and 7 unaffected individuals in the family.

## 3. Results

### 3.1. Clinical Features

The demographic and clinical features of the living affected members and mutation carriers are summarized in Table 1. The age of enrollment ranged from 1 to 82 years. The visual acuity ranged from 20/30 to nonlight perception (NLP). Seven family members had refractive errors, including myopia (ranging −0.75 to −8 diopters) and astigmatism, and twelve members presented with cataract. All affected individuals except for the proband had congenital night blindness, and seven affected members already presented legal blindness. The proband's mother (V:1) and aunt (V:3) had breast cancer, and his father (V:2) had fatty liver disease.

### 3.2. Mutations in the CYP4V2 Gene

Two previously reported *CYP4V2* mutations (c.802-8_810del17insGC and c.992A>C (p.H331P)) were detected in this family. The proband (VI:1) was compound heterozygous for both mutations. The c.802-8_810del17insGC mutation was maternally derived (V:1), whereas the c.992A>C mutation was paternally inherited (V:2). Two other family members (V:9 and VII:1) were heterozygous for the c.802-8_810del17insGC mutation.

### 3.3. Mutations in the PRPF3 Gene

**Figure 2:**
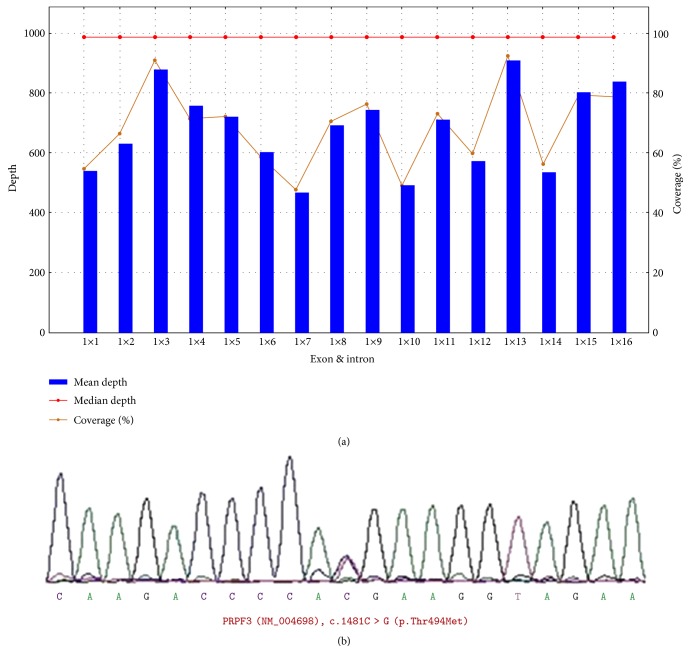
The depth and coverage of next-generation sequencing of the *PRPF3* gene and the chromatogram obtained by Sanger sequencing (patient III:9). (a) The rectangle shows the averaged sequencing depth and coverage of the family for all 16 exons of the *PRPF3* gene as screened by next-generation sequencing. (b) Sanger sequencing detected a heterozygous mutation (c.1481C>T, p.Thr494Met) in *PRPF3*.

Targeted NGS of two affected members (IV:1 and III:9) revealed one common missense mutation in the *PRPF3* gene (c.1481C>T) (Figure 2), which was then screened by Sanger sequencing in 8 affected (III:9, IV:1, IV:3, IV:6, IV:14, V:9, V:12, and VI:1) and seven unaffected family members (IV:9, V:1,V:7, V:11, V:16, V:20, and VI:13) for cosegregation analysis.

### 3.4. Clinical and Molecular Manifestations of Affected Family Members

Two types of clinical and molecular manifestations were observed in this family: (i) a BCD phenotype that was related to the compound heterozygous *CYP4V2* mutations and (ii) a RP phenotype that was associated with the *PRPF3* mutation and followed an autosomal dominant pattern of inheritance.

#### 3.4.1. Type 1 (Proband VI:1)

**Figure 3:**
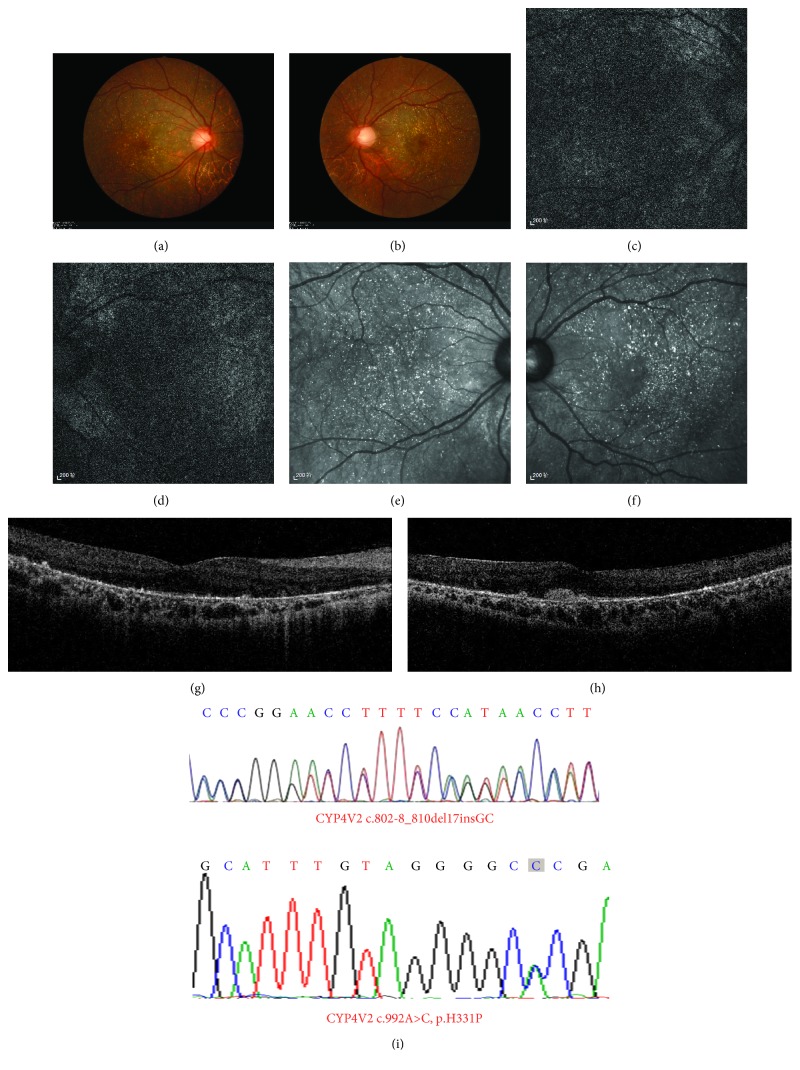
Fundal images and chromatograms of the proband with a clinical diagnosis with Bietti's crystalline corneoretinal dystrophy and harboring compound heterozygous mutations in the *CYP4V2* gene (patient VI:1). Fundal photographs (a, b), autofluorescence images (c, d), and near-infrared images (e, f) of both eyes are shown on the left, and chromatograms of two mutations are demonstrated on the right (g, h).

The proband was a 33-year-old man referred to us for genetic counseling based on a significant decrease in visual acuity starting at the age of 17 years. The patient developed night blindness in his early 30s. He had high myopia (−7.00 D) in both eyes, and best-corrected Snellen visual acuity was 20/30 in his both eyes. There was a history of chronic uveitis in his left eye since age 28. He was diagnosed with BCD based on clinical findings that included numerous tiny glistening yellow-white crystals scattered at the posterior pole of the retina, RPE atrophy (Figure 3), and decreased responses in FERGs and mfERGs.

Two previously reported disease-causing mutations in *CYP4V2* (c.802-8_810del17insGC in exon 7 and c.992A>C (p.H331P) in exon 8) were identified in the proband [19]. The compound heterozygosity was confirmed by screening his unaffected parents; his mother (V:1) carried the c.802-8_810del17insGC variant, and his father (V:2) harbored the c.992A>C mutation. The proband's unaffected son (VII:1) had the c.802-8_810del17insGC mutation, whereas no pathogenic *CYP4V2* mutations were detected in the apparently normal daughter (VII:2). Notably, no *PRPF3* mutations were detected in the proband.

#### 3.4.2. Type 2

**Figure 4:**
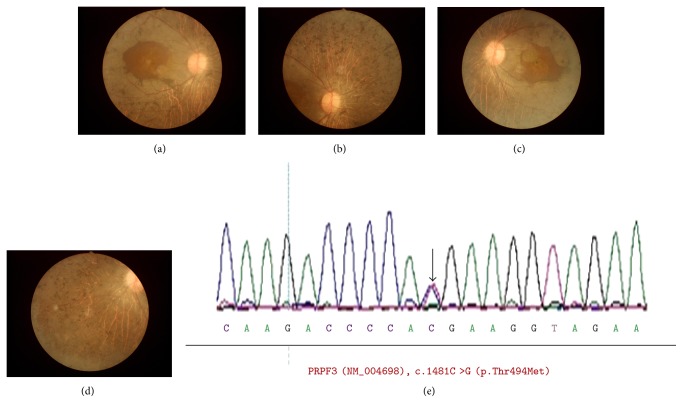
Fundal photographs and chromatogram of a patient with severe phenotype of retinitis pigmentosa and harboring *PRPF3* mutations (patient IV:12). Fundal photographs of the right eye (a, b) and the left eye (c, d), and chromatogram of the *PRPF3* mutation (c.1481C>T, p.T494M) is shown (e).

In addition to the proband, other family members affected with adRP presented with night blindness since birth. Best-corrected visual acuity was from 200/400 to NLP. Fundus examination showed severe features of RP, with a mass of bone-spicule pigmentation depositions, more severe RPE atrophy involving the macular and choroidal sclerosis extending to the midperipheral retina, whereas partial attenuation of the retinal blood vessels, slight waxy pallor of the optic disc, was presented (Figure 4). FERG demonstrated undetectable responses both in scotopic and photopic conditions and extinguished mfERG.

One *PRPF3* mutation, c.1481C>T (p.T494M), was detected in 13 family members, including 11 males and 2 females. No novel mutation and previously reported mutations were detected in the other 45 genes in the panel. The identified mutation (c.1481C>T) cosegregated with the RP phenotype in 11 affected family members tested and was not observed in 9 unaffected family members (Figure 1). This mutation was observed across four generations. Taken together, the c.1481C>T mutation was considered to be the main cause of adRP in this family.

## 4. Discussion

*PRPF3* (MIM 607301) is a precursor mRNA-processing factor gene that was first identified for adRP in 2002 [20]. In the present study, a pathogenic mutation (c.1481C>T, p.T494M) in the *PRPF3* gene was identified in 11 individuals presenting an adRP phenotype in a five-generation Chinese family. The molecular genetic features of a Chinese pedigree with a *PRPF3* mutation have been previously reported. The c.1481C>T mutation is considered to be one of the most common mutations in *PRPF3* [20–25]. Previous reports have shown that patients harboring the c.1481C>T mutation develop early-onset night blindness, visual field loss, and visual acuity loss between the ages of 30 and 40, as well as loss of ERG responses after the age of 30. Compared to those in previously reported Japanese, Spanish, Korean, Swiss, and North American families, members of this Chinese family with the c.1481C>T mutation presented a more severe disease phenotype, which included congenital blindness, severe visual acuity loss, extended RPE atrophy, and completely extinguished ERG responses.

Mutations in the *CYP4V2* gene (MIM 608614) are the only known causative factor for BCD to date. The *CYP4V2* gene consists of 11 exons and encodes a 525 amino acid protein belonging to the *CYP450* family. *CYP4V2* is widely expressed in tissues, including the retina, RPE, lymphocytes, heart, brain, placenta, lung, liver, skeletal muscle, kidney, and pancreas, which has been thought to play a crucial role in fatty acid and corticosteroid metabolism. In the present study, two compound heterozygous mutations in *CYP4V2* (c.802-8_810del17insGC and c.992A>C) were identified in the proband who presented typical BCD. In our previous study, *CYP4V2* mutation screening among 92 Chinese patients with BCD showed that c.802-8_810del17insGC and c.992A>C are common pathogenic mutations in Chinese with BCD [26]. The parents of the proband are not a consanguineous marriage couple. So we speculate that these heterozygous mutations in Chinese population may be universal. This phenomenon may be related to the common ancestor based on the huge population of China. The heterozygous state of the same gene carried by parents is consistent with the autosomal recessive inheritance pattern. This will be important for prenatal testing for family planning, early finding carrier status, and determining risk of inheritance in Chinese.

Coexistence of variants in two or three genes associated with retinal degeneration has rarely been reported in a family [3]. In the present study, we identified the coexistence of two distinct phenotypes in one family, namely, BCD and RP, which were caused by the pathogenic variants in the *CYP4V2* and *PRPF3* genes, respectively. The mode of inheritance of the two diseases was maintained in this family, in which BCD demonstrated an autosomal recessive trait and RP showed an autosomal dominant trait.

Two types of clinical and molecular manifestations identified in this study include (i) a BCD phenotype related to *CYP4V2* mutations and (ii) an RP phenotype related to *PRPF3* variants. Clinical features for (i) BCD and (ii) RP of this family were similar to those in previous reports. The proband affected with BCD in this family had a later onset for night blindness and relatively slow progression, with a predominantly affected choroid at the posterior pole. On the other hand, family members with RP caused by the *PRPF3* mutation showed a more severe phenotype. Our study provides a better understanding of the genotype-phenotype correlation in a family with two independent pathogenic gene mutations and may be used in clinics for the differential diagnosis of retinal degenerations.

In summary, this is the first report on *PRPF3*-associated adRP and *CYP4V2*-associated arBCD in a large multigenerational Chinese family. The inheritance pattern of each gene mutation is independent. Our study provides an insight into the clinical effects of two independent gene mutations in a large family to facilitate accurate diagnosis and disease counseling.

## Figures and Tables

**Table 1:** Demographic and clinical features and genotypes of *PRPF3* and *CYP4V2* mutations in a family with adRP and arBCD.

Number	Gender	Age	First symptom	Phenotype	BCVA		Eye complications	Systemic diseases	*PRPF3* mutation	*CYP4V2* mutations	
					OD	OS			c.1481C>G	c.802-8_810del17insGC	c.992A>C
III:8	Female	76	None	Normal	20/250	20/250	Cataract		ND	ND	ND
III:9	Male	80	Night blindness	RP	LP	LP	Cataract		MT	Wild	Wild
III:10	Female	80	None	Normal	20/50	20/50	Cataract		ND	ND	ND
IV:1	Female	82	Night blindness	RP	LP	LP	Cataract		MT	Wild	Wild
IV:3	Male	57	Night blindness	RP	LP	LP	Cataract		ND	Wild	Wild
IV:5	Male	57	Night blindness	RP	LP	LP	Cataract		ND	ND	ND
IV:6	Male	57	Night blindness	RP	LP	LP	Cataract		MT	Wild	Wild
IV:7	Female	54	None	Normal	20/30	20/30	None		Wild	Wild	Wild
IV:10	Male	39	Night blindness	RP	20/200	20/200	None		MT	Wild	Wild
IV:12	Male	35	Night blindness	RP	20/400	LP	None		ND	Wild	Wild
IV:14	Male	55	Night blindness	RP	20/100	20/200	Cataract		MT	Wild	Wild
IV:15	Female	50	None	Normal	20/35	20/35	None		ND	ND	ND
IV:16	Female	51	None	Normal	20/35	20/20	None		ND	ND	ND
IV:18	Male	49	None	Normal	20/20	20/20	None		ND	ND	ND
IV:20	Male	45	Night blindness	RP	20/100	20/200	None		MT	Wild	Wild
V:1	Female	57	None	Normal	20/20	20/25	None	Breast cancer	ND	MT	Wild
V:2	Male	60	None	Normal	20/30	20/30	Cataract	Fatty liver disease	ND	Wild	MT
V:3	Female	55	Night blindness	RP	20/10	20/200	None	Breast cancer	MT	Wild	Wild
V:7	Male	45	None	Normal	20/30	20/30	Myopia		Wild	Wild	Wild
V:8	Female	46	None	Normal	20/18	20/18	None		Wild	Wild	Wild
V:9	Male	43	Night blindness	RP	20/200	LP	Myopia		MT	Wild	Wild
V:11	Male	24	None	Normal	20/18	20/18	None		Wild	Wild	Wild
V:12	Male	22	Night blindness	RP	20/100	20/100	Myopia		MT	Wild	Wild
V:13	Male	13	None	Normal	20/30	20/20	None		ND	Wild	Wild
V:15	Female	8	None	Normal	20/20	20/20	None		ND	Wild	Wild
V:17	Male	37	None	Normal	20/25	20/25	None		ND	Wild	Wild
V:18	Female	35	None	Normal	20/18	20/18	High myopia		ND	ND	ND
V:19	Female	35	None	Normal	20/25	20/20	Myopia		ND	ND	ND
V:20	Male	33	None	Normal	20/18	20/18	None		Wild	Wild	Wild
V:31	Male	15	None	Normal	20/18	20/18	Myopia		ND	ND	ND
VI:1	Male	33	Decreased vision	BCD	20/25	20/30	Myopia, uveitis		Wild	MT	MT
VI:2	Female	29	None	Normal	20/40	20/50	High myopia		ND	ND	ND
VI:10	Female	22	None	Normal	20/18	20/18	Myopia		ND	Wild	Wild
VI:12	Male	15	None	Normal	20/20	20/20	None		ND	Wild	Wild
VI:14	Male	13	None	Normal	20/20	20/20	None		ND	Wild	Wild
VI:15	Male	10	None	Normal	20/20	20/20	None		ND	Wild	Wild
VI:16	Male	9	None	Normal	20/20	20/20	None		ND	Wild	Wild
VII:1	Male	5	None	Normal	20/30	20/30	None		ND	MT	Wild
VII:2	Female	1	None	Normal	20/30	20/200	Hyperopia		ND	Wild	Wild

MT: mutation; ND: not detected; LP: light perception; NLP: nonlight perception.
